# Intervention meta-analysis: application and practice using R software

**DOI:** 10.4178/epih.e2019008

**Published:** 2019-03-28

**Authors:** Sung Ryul Shim, Seong-Jang Kim

**Affiliations:** 1 Department of Preventive Medicine, Korea University College of Medicine, Seoul, Korea; 2 Urological Biomedicine Research Institute, Soonchunhyang University Hospital, Seoul, Korea; 3 Department of Nuclear Medicine, Pusan National University Yangsan Hospital, Pusan National University School of Medicine, Yangsan, Korea; 4 BioMedical Research Institute for Convergence of Biomedical Science and Technology, Pusan National University Yangsan Hospital, Yangsan, Korea

**Keywords:** Meta-analysis, Meta-regression, Forest plot, Heterogeneity, Publication bias, R software

## Abstract

The objective of this study was to describe general approaches for intervention meta-analysis available for quantitative data synthesis using the R software. We conducted an intervention meta-analysis using two types of data, continuous and binary, characterized by mean difference and odds ratio, respectively. The package commands for the R software were “metacont”, “metabin”, and “metagen” for the overall effect size, “forest” for forest plot, “metareg” for meta-regression analysis, and “funnel” and “metabias” for the publication bias. The estimated overall effect sizes, test for heterogeneity and moderator effect, and the publication bias were reported using the R software. In particular, the authors indicated methods for calculating the effect sizes of the target studies in intervention meta-analysis. This study focused on the practical methods of intervention meta-analysis, rather than the theoretical concepts, for researchers with no major in statistics. Through this study, the authors hope that many researchers will use the R software to more readily perform the intervention meta-analysis and that this will in turn generate further related research.

## INTRODUCTION

Meta-analysis systematically and objectively selects target literature, quantifies the results of individual studies, and provides the overall effect size thus enabling the right decision making for evidence-based medicine [1-5].

Computer software applications that facilitate the approach to meta-analysis include STATA, R, SAS, MIX, comprehensive meta-analysis (CMA), Review Manager (RevMan), and Meta-Analyst.

RevMan and CMA are adequate for beginners because they provide a graphic user interface, but their expandability is limited because they do not provide support for specific topics such as network meta-analysis, diagnostic test meta-analysis, dose-response meta-analysis, and genome meta-analysis.

In contrast, R and STATA have excellent expandability and support various analytical methods developed thus far ranging from intervention meta-analysis to diagnostic test meta-analysis.

STATA is a standard and reliable commercial application with excellent expandability as most statistical modules are verified in the *STATA Journal*.

R is a free application and supports a programming language for statisticians. While it does require a fair amount of learning, anyone can readily use R once they understand the basics of using packages, datasets and functions. Moreover, R Studio is very convenient to use as it offers a graphic user interface.

In this paper, the previous meta-analysis studies performed by authors [1-3] are reviewed using the R software. Furthermore, this work considers the types and changes in effect sizes for calculating the overall effect size from the beginning, so it requires prior knowledge on the preliminary processes for meta-analysis such as systematic literature collection based on PICO (population, intervention, comparison, outcome), data extraction, and quality assessment.

## UNDERSTANDING THE EFFECT SIZE

One should first understand the effect size before performing meta-analysis. In simple terms, the effect size is the impact of a specific intervention. One example is the benefit (or deterioration) tied to a specific medicine or therapy. The effect size is usually expressed as a quantitative value [1-3].

The effect size is expressed as a difference in means in continuous data, or as an odds ratio (OR), relative risk (RR), or hazard ratio for binary data or time to event data, and as a percentage for proportion or rate data. However, the correlation coefficient (r) between variables is not generally used as effect size because the specific effect size of treatments is considered important than correlation coefficient in healthcare meta-analysis.

The OR and percentage can readily be used as effect size as they are already standardized across various datasets.

However, the standardization of the effect size should be considered when the difference in means is used as the effect size for continuous variables. In Cochrane, the mean difference (MD) or difference in means can be used when individual studies use the same scale; it is straightforward to interpret as the unit can be accepted as is. Weighted mean difference or absolute mean difference is also same meaning with MD.

The standardized mean difference (SMD) must be standardized for comparison if the individual studies use different units. Standardization here implies dividing each effect size by the underlying standard deviation (SD) [4,5].

**Figure 1.**
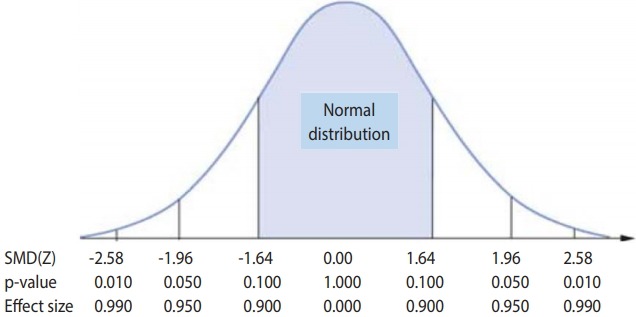
Effect size of standardized mean difference (SMD).

Figure 1 provides a good illustration of the SMD, which is equal to the probability density corresponding to the z-value of a standard normal distribution curve. For example, if the SMD is 1.96, it is located at 47.5% in the positive direction. It is worth noting that the SMD must be interpreted in a single direction according to the direction of the effect size, because the reference point of the reference group is zero. Therefore, an interpretation of a SMD of 1.96 is that “the treatment group is 95.0% better or poorer than the reference group.”

### Calculating the effect size

This study addresses the calculation of the effect size and the standard error (SE), which both need to be understood for metaanalysis. An application-based study reveals that meta-analysis can help calculate the overall effect size using the raw data itself, and also obtain the overall effect size by using the summarized effect size and SE as well.

Thus, it is critical to calculate the effect size and SE in individual studies, and one must be able to compute them before approaching the specific topics of meta-analysis below.

#### Continuous data example

For a treatment group and a control group, m1 is the difference between the means before treatment (pre_mean1) and after treatment (post_mean1) in the treatment group, and m2 is the difference between the means before and after treatment in the control group. Thus, s1 and s2 are the SDs of m1 and m2, respectively.

“MD” is simply the difference in means between the treatment and control groups (m1-m2, the direction of MD, is set as intended by the researcher), and the pooled SD and SE are calculated as follows:

$$SD=\sqrt{\frac{\left(n_{1}-1\right)s_{1}^{2}+\left(n_{2}-1\right)s_{2}^{2}}{n_{1}+n_{2}-2}},\;SE=SD\;*\;\sqrt{\frac{1}{n_{1}}+\frac{1}{n_{2}}}$$

The SMD is determined by dividing MD by the pooled SD (SMD=MD/SD), and the SE equals SD multiplied by the square root of the sum of the inverse of each number of samples.

The SMD here is Cohen’s d. If the number of samples is small, the overall effect size tends to be overestimated. To correct this, Hedges’ g is used.

#### Binary data example

The binary data example is often expressed as a 2×2 table according to the existence or absence of a treatment and a disease. We consider the following elements in the table: there is treatment and the condition improves (true positive, tp), there is treatment and the condition does not improve (false positive, fp), there is no treatment and the condition improves (false negative, fn ), and there is no treatment and the condition does not improve (true negative, tn). The effect size OR and SE can be calculated as follows:

$$\mathrm{OR}=\frac{\left(\mathrm{tp}*\mathrm{tn}\right)}{\mathrm{fp}*\mathrm{fn}},\;SE=\sqrt{\frac{1}{\mathrm{tp}}+\frac{1}{\mathrm{fp}}+\frac{1}{\mathrm{fn}}+\frac{1}{\mathrm{tn}}}$$

### INTERVENTION META-ANALYSIS USING THE R “meta” PACKAGE

**Figure 2.**
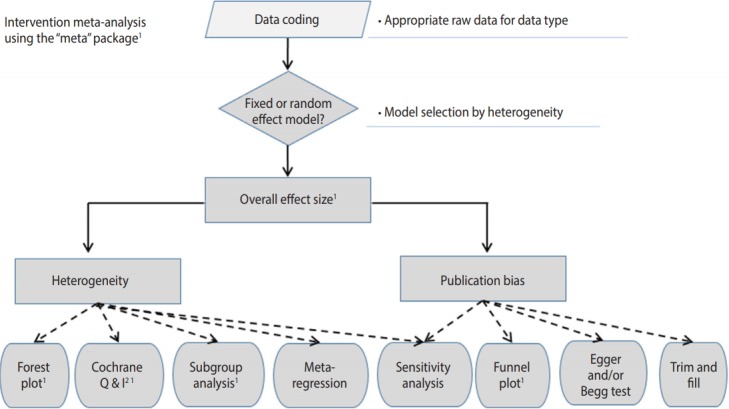
Flow chart of intervention meta-analysis using R "meta" package. ^1^ Recommend.

Figure 2 shows the general flow for intervention meta-analysis. First, in data encoding, variable names must be modified in accordance with the corresponding function. Next, a meta-analysis model is selected (fixed or random), the overall effect size is presented, the heterogeneity is checked, and the publication bias is checked and reported.

The R packages for meta-analysis largely include “meta”, “metafor”, and “rmeta.” They both have strengths and weaknesses. They should be installed in advance as follows [6]:

· install.packages(“meta”)

· install.packages(“metafor”)

· install.packaqes(“rmeta”)

Key explanations will be provided regarding the “meta” package, which is generally easy to use. We mark R commands with a dot (‘· ’) in front of them, to distinguish them from the main text. When long commands are extended to the next line, there is no dot at the beginning of the next line. Thus, when you enter the command in the R software, you must type them without the dot (‘· ’) in front of them.

#### Continuous data example

##### Data encoding and loading

A study on the meta-analysis of the effect of stem cell therapy on the bladder function in an animal model with a spinal cord injury is used as an example, and the voiding pressure is used as the result index. The total number of studies was 11, comprising 94 subjects in the experimental group and 93 subjects in the control group. The subgroup 1 is the contusion model and the subgroup 0 is the transection and hemisection model (Supplementary Material 1).

We first load the “meta” package to perform the meta-analysis:

· library(meta)

Then, we load the example file to the R memory from the working folder with the following command. It is worth noting that R prefers the comma-separated values (csv) file format. Thus, you should save Supplementary Material 1 as “shim_con.csv” in the specified working folder.

·data_con <- read.csv(“shim_con.csv”, header=TRUE)

“read.csv” is a function for loading a csv file. The above command implies loading the file “shim_con.csv” and use the first variable name (header=TRUE) as is. This loaded file is saved as “data_con” in the R memory.

##### Overall effect size

The meta package includes various functions. Among them, the “metacont” function calculates the overall effect size when all the raw data consists of continuous data as follows:

· ma_con <- metacont(n1, m1, s1, n2, m2, s2, sm=“SMD”, method.smd=“Hedges”, study, byvar=g ,data=data_con)

· print(ma_con, digits=3)

For continuous data, we sequentially enter the number of samples, mean, and SD of the treatment group and the control group; alternatively we can enter them the in opposite direction as intended.

To calculate the effect size without standardization when the individual studies have the same unit, smd=“MD” can be entered. However, the standardized effect size is usually expressed as the SMD, and there are several methods to calculate it. The most basic method is Cohen’s d which is determined by dividing the effect size by the SD. However, it tends to overestimate the overall effect size when the number of samples is small. Thus, the use of Hedges’ g is recommended to correct it (method.smd=“Hedges” or “Cohen”). Hedges’ g is calculated by multiplying the correction index J by Cohen’s d.

$$J=1-\frac{3}{4\left(n_{1}+n_{2}\right)-9}$$

To select a fixed or random effect model, enter comb.fixed=TRUE or FALSE, and comb.random= TRUE or FALSE. If no model is selected, the metacont function shows the results for both models.

**Figure 3.**
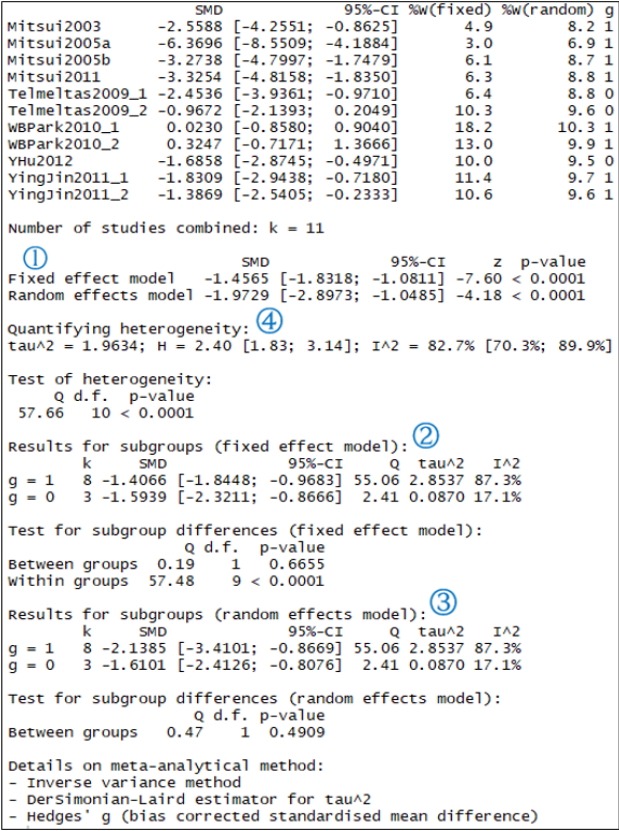
Overall effect size of continuous example. SMD, standardized mean difference; CI, confidence interval; g, subgroup.

“study” represents the name of individual studies, and “data=data_con” assigns the “data_con” data loaded in the R memory. “byvar=g” is entered to show the result of each subgroup, where g is a variable name representing a subgroup. The results of the “metacont” function are assigned to ma_con, and are shown in Figure 3.

In the following, we examine the results in ma_con, shown in Figure 3, one by one.

① indicates the overall effect size of all 11 studies. The SMD of the fixed effect model is -1.456 (95% confidence interval [CI], -1.831 to -1.081) and the p-value is lower than 0.0001, indicating that the treatment achieved a statistically significant improvement. The SMD of the random effect model is -1.973 (95% CI, -2.897 to -1.048) and the p-value is lower than 0.0001, pointing to an identical result.

② and ③ indicate the result corresponding to the subgroup as a fixed and random effect model. In the random model, a difference between the subgroups (0 vs. 1) is suspected.

④ shows the heterogeneity of all studies. The Higgins’ I^2^ of heterogeneity is determined by subtracting the degrees of freedom from the Cochrane Q statistics and then dividing the resulting value by the Cochrane Q statistics again. Thus, heterogeneity is quantified in a consistent manner. Values between 0% and 40% indicate that heterogeneity might not be very pronounced; values between 30% and 60% indicate moderate heterogeneity, values between 50% and 90% indicate substantial heterogeneity, and values between 75% and 100% point to considerable heterogeneity. The significance of the Cochrane Q statistics is based on the p-value of 0.1, which is a slightly wide range [4]. In this continuous data example, the Higgins’ I^2^ is 82.7% and the p-value of the Cochrane Q statistics is lower than 0.0001, which suggests the existence of heterogeneity. Therefore, the random effect model must be given priority in the aggregate model.

In addition, the calculation process for the results is indicated at the bottom of Figure 3. Inverse variance is a basic meta-analysis method that uses the inverse variance of the relevant study to calculate the weights of individual studies. The DerSimonian-Laird estimator (DL) is a method for using the tau value to calculate the between-study variance in the random effect model. Hedges’ g means that the current result used the Hedges’ g correction to the Cohen’s d.

You can freely adjust the detailed calculation method while referring to the “meta” package.

#### Forest plot

**Figure 4.**
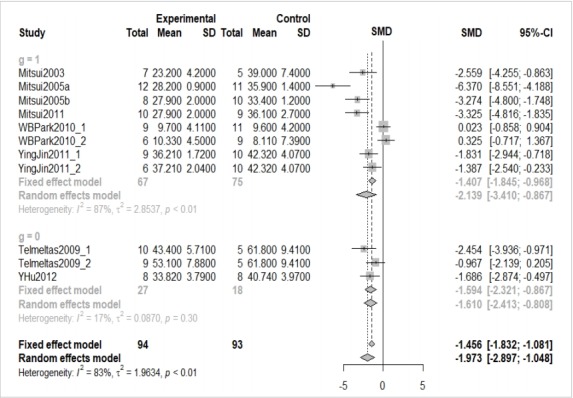
Forest plot of continuous example. SD, standard deviation; SMD, standardized mean difference; CI, confidence interval; g. subgroup .

Figure 3 is effective in providing detailed information, but its overall readability is low. Therefore, the understanding of readers can be improved by drawing a forest plot as follows (Figure 4).

· forest(ma_con, comb.fixed=TRUE, comb.random=TRUE,digits=3,rightcols=c(“effect”, “ci”))

We enter the corresponding meta-analysis model (ma_con) in the “forest” function. Then we can enter various options to enhance the appearance of the graph. “comb.fixed=TRUE” and “comb.random=TRUE” can be used to display both models, “digits=3” is used to round down the display to only three decimal places, and “rightcols=c(“effect”,”ci”))” is used to show the effect size and 95% CI only while omitting the weight on the right side of the forest plot.

Figure 4 provides the same information as the overall effect size mentioned above. Furthermore, within-study and between-study variations can readily be identified through the graphic representation of the effect size of individual studies.

For example, it can be seen that there are large within-study variations in Mitsui2005_and Mitsui2003, while there are large betweenstudy variations in Mitsui2005_a, WBPark2010_1 and WBPark2010_2.

##### Heterogeneity

To properly interpret the overall effect size obtained from metaanalysis, the existence or absence of heterogeneity between studies should be checked and any significant moderator should be tested and reported. There are many potential causes for this heterogeneity, such as chance, differences in study design, research environment, and the demographic factors of the sample population.

#### Visual verification: forest plot and subgroup analysis

Within-study and between-study variations can easily be verified visually to explore heterogeneity.

#### Measuring heterogeneity: Higgins’ I^2^ and Cochrane Q statistics p-value

The heterogeneity of the studies was explained in detail with respect to Figure 3. The degree of heterogeneity is quantified, and the statistical test is shown as well.

#### Identifying the cause of heterogeneity: meta-regression

If heterogeneity is doubted during the visual verification using a forest plot and the heterogeneity values using the Cochrane Q statistics and Higgins’ I^2^, meta regression analysis can be performed to statistically test and identify the cause of heterogeneity, as follows:

·metareg(ma_con,g, method.tau=”REML”, digits=3)

The selected metal-analysis model must be entered in the “metareg” function, and the method.tau=”REML”(restricted maximum-likelihood estimator or “ML”(maximum-likelihood estimator), or “DL”(DerSimonian-Laird estimator) must be selected in accordance with the method of assigning weights to meta regression analysis. The values may be changed depending on the weight calculation method, but the statistical direction is almost same.

Based on the random effect model, the overall effect size of subgroup 1 is -2.139 (95% CI, -3.410 to -0.867) and the overall effect size of subgroup 0 is -1.610 (95% CI, -2.413 to -0.808). Thus, this variable seemed to be a moderator, but the result of the meta regression analysis pointed to its statistical insignificance, with p-value=0.711.

· bubble(metareg(ma_con, g, method.tau=”REML”))

**Figure 5.**
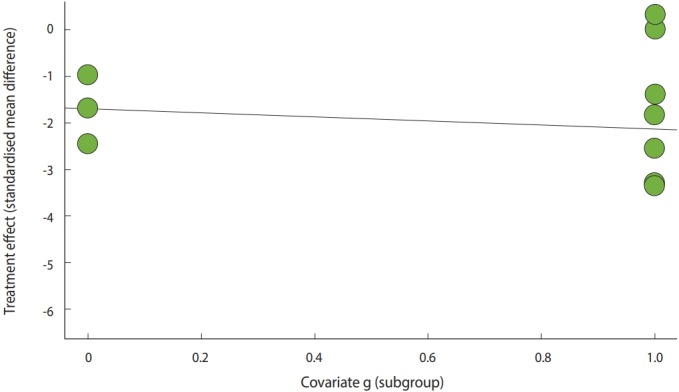
Meta-regression bubble plot of continuous example.

The result of the meta regression analysis can be plotted as shown in Figure 5. The straight line on this graph indicates a regression line, and the statistical test for the slope of this line is the p-value of the meta regression analysis mentioned above.

##### Checking publication bias

The publication bias means the error in connection with whether a study is published or not depending on the characteristics and result of individual studies. This error is caused because statistically significant study results generally have a higher likelihood of being published. One should check whether the result of the meta-analysis is overestimated or underestimated considering this publication bias.

#### Visual verification: funnel plot

**Figure 6.**
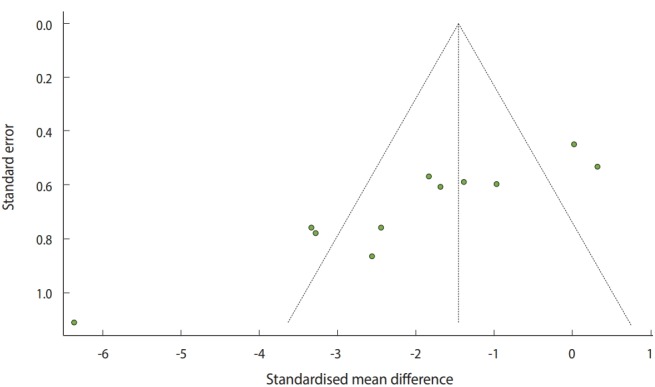
Funnel plot of continuous example.

To examine the publication bias, the existence of asymmetry between studies needs to be verified visually (Figure 6) as follows:

· funnel(ma_con, comb.fixed=TRUE, comb.random=FALSE)

The selected metal-analysis model must be entered in the funnel function and comb.fixed=TRUE or FALSE, and comb.random=TRUE or FALSE must be entered subsequently.

The y-axis of the funnel plot indicates the sample size (SE) and the x-axis indicates the effect size. In general, small-scale studies are distributed widely at the bottom, whereas large-scale studies are distributed narrowly at the top within the funnel. Therefore, when the studies are distributed symmetrically at the top within the funnel, the publication bias can be considered to be small.

For the continuous data example, there are three studies on the left and two studies on the right outside the funnel, and four studies on the left and two studies on the right within the funnel. Thus, it is apparent that a publication bias exists (Figure 6).

#### Statistical test of publication bias

General methods to statistically test the publication bias include Egger’s linear regression method test (Egger’s test) and Begg and Mazumdar’s rank correlation test (Begg’s test). It has been reported that Egger’s test estimates the effect size more accurately than Begg’s test. However, Cochrane does not recommend a statistical test because the accuracy of the test is more sensitive to the number of small studies (small study effect) than to the publication bias.

■ Egger’s linear regression method test

Egger’s test represents as a regression equation the relationship between the SE of the intervention effect and the effect size of individual studies. The null hypothesis in Egger’s test is that the slope of the linear regression model is zero. If the null hypothesis cannot be rejected, there is no evidence of publication bias.

· ma_con <- metacont(n1, m1, s1, n2, m2, s2, sm=”SMD”, method.smd=”Hedges”, study,data=data_con)

·metabias(ma_con, method.bias=”linreg”)

It is likely that the “metabias” function will not be carried out because the overall effect size is calculated separately for each subgroup. Therefore, the overall effect size for all studies is calculated once again and then the “metabias” function is used immediately. The selected meta-analysis model must be entered in the “metabias” function with the option method.bias=”linreg” to perform Egger’s test.

For continuous data, the coefficient of the bias is -9.23, indicating the initial value (intercept) and the p-value is lower than 0.0001. Thus, the null hypothesis is rejected, and the existence of a publication bias can be confirmed.

■ Begg and Mazumdar’s rank correlation test

The correlation between the standardized effect size and the SE of individual studies is tested through corrected rank correlation. An insignificant rank correlation test is consistent with the absence of publication bias.

·metabias(ma_con, method.bias=”rank”)

The selected metal-analysis model must be entered in the metabias function with the option method.bias=”rank” for performing Begg’s test.

As with the result of Egger’s test, the p-value is 0.0024, indicating that there is a publication bias.

Thus, when the publication bias is statistically significant, one needs to verify the overall effect size once again by including and excluding studies that appear to hold a publication bias. In other words, sensitivity analysis should be performed for publication bias and to report the characteristics of the studies. If heterogeneity is found, a statistical test should also be performed through meta regression analysis.

### Binary data example

The commands for meta-analysis are almost identical to those in the continuous data example. Refer to the reference [2] and appedix commands for detailed descriptions for binary data (Supplementary Material 2).

### Meta-analysis regardless of data type

So far we have examined the overall effect size in raw data from continuous data and binary data and methods to evaluate related heterogeneity.

However, this delineation in the data is simply linked to a delineation in commands (functions) for user convenience. If the effect size and SE of individual studies are already known, metaanalysis can be performed regardless of the data type.

#### Data encoding and loading

When the raw data from continuous and binary data (Supplementary Material 1) is loaded, the effect size and SE are already input as variables.

·data_con <- read.csv(“shim_con.csv”, header=TRUE)

·data_bin <- read.csv(“hwang_bin.csv”, header=TRUE)

#### Overall effect size

The meta package includes several functions. Among them, the “metagen” function calculates the overall effect size by effect size and SE of individual studies.

### Calculating the effect size and standard error for continuous data

· ma_con_es <- metagen(cohen_d, cohen_se, sm=”Cohen(SMD)”, study, byvar=g, data=data_con)

· print(ma_con_es, digits=3)

· forest(ma_con_es, comb.fixed=TRUE, comb.random=TRUE, digits=3, rightcols=c(“effect”, “ci”))

Cohen_d, and cochen_se, respectively the effect size and the SE in the metagen function, must be entered. The meta-analysis model determined with the effect size and SE of continuous data is saved as ma_con_es.

In chapter 1 of continuous data example mentioned above, if Cohen’s d is used as an option for SMD [method.smd=”Cohen”], we can obtain the same effect size as the meta-analysis model ma_con_es that was just calculated.

### Calculating the effect size and standard error of binary data

· ma_bin_es <- metagen(lnor, orse, sm=”OR”, study, data=data_bin)

· print(ma_bin_es, digits=3)

· forest(ma_bin_es, comb.fixed=TRUE, comb.random=TRUE, digits=3, rightcols=c(“effect”, “ci”))

In “ma_bin_es” data, lnor and orse variable, respectively the effect size and the SE, must be entered in the “metagen” function. The meta-analysis model determined by the effect size and the SE of the binary data is set to ma_bin_es.

It is apparent that this is identical to the effect size In chapter 2 of binary data example mentioned above.

## CONCLUSION

This study de-emphasized statistical theory and instead focused on the actual performance of meta-analysis so that researchers with no statistics major can easily perform meta-analysis.

Researchers interested in performing meta-analysis by referring to this study should firmly establish the concepts for effect size calculation. We hope that this study will facilitate the use of R by researchers to perform meta-analysis and generate related research.

## SUPPLEMENTARY MATERIALS

Korean version is available at http://www.e-epih.org/.
