# Supplementary material for: Intervention meta-analysis: application and practice using R software
Source: Epidemiol Health. 2019 Mar 28;41:e2019008. doi: 10.4178/epih.e2019008 (PMC6545497; doi:10.4178/epih.e2019008)
Supplement: Supplementary file 1 [file epih-41-e2019008-supplementary.pdf]

# Supplementary Material 1. Sample data

Table S1. The sample data for continuous outcomes

| study | n1 | m1 | s1 | n2 | m2 | s2 | md | sd | se | cohen_d | cohen_se | g |
|---|---|---|---|---|---|---|---|---|---|---|---|---|
| Mitsui2003 | 7 | 23.2 | 4.2 | 5 | 39.0 | 7.4 | -15.8 | 5.7 | 3.337 | -2.772 | 0.814 | 1 |
| Mitsui2005a | 12 | 28.2 | 0.9 | 11 | 35.9 | 1.4 | -7.7 | 1.2 | 0.486 | -6.608 | 1.060 | 1 |
| Mitsui2005b | 8 | 27.9 | 2.0 | 10 | 33.4 | 1.2 | -5.5 | 1.6 | 0.759 | -3.438 | 0.744 | 1 |
| Mitsui2011 | 10 | 27.9 | 2.0 | 9 | 36.1 | 2.7 | -8.2 | 2.4 | 1.082 | -3.481 | 0.728 | 1 |
| Telmeltas2009_1 | 10 | 43.4 | 5.7 | 5 | 61.8 | 9.4 | -18.4 | 7.1 | 3.866 | -2.607 | 0.726 | 0 |
| Telmeltas2009_2 | 9 | 53.1 | 7.9 | 5 | 61.8 | 9.4 | -8.7 | 8.4 | 4.697 | -1.033 | 0.591 | 0 |
| WBPark2010_1 | 9 | 9.7 | 4.1 | 11 | 9.6 | 4.2 | 0.1 | 4.2 | 1.870 | 0.024 | 0.449 | 1 |
| WBPark2010_2 | 6 | 10.3 | 4.5 | 9 | 8.1 | 7.4 | 2.2 | 6.4 | 3.391 | 0.345 | 0.531 | 1 |
| YHu2012 | 8 | 33.8 | 3.8 | 8 | 40.7 | 4.0 | -6.9 | 3.9 | 1.941 | -1.783 | 0.591 | 0 |
| YingJin2011_1 | 9 | 36.2 | 1.7 | 10 | 42.3 | 4.1 | -6.1 | 3.2 | 1.465 | -1.917 | 0.555 | 1 |
| YingJin2011_2 | 6 | 37.2 | 2.0 | 10 | 42.3 | 4.1 | -5.1 | 3.5 | 1.799 | -1.467 | 0.578 | 1 |

n1, treatment group sample size; m1, treatment group mean; s1, treatment group standard deviation; n2, control group sample size; m2, control group mean; s2, control group standard deviation; md, mean difference; sd, pooled sd; se, pooled standard error; cohen_d, SMD; cohen_se, standard error of SMD; g, group. Shim SR et al.[2].

Table S2. The sample data for binary outcomes

| study | tp | fp | fn | tn | or | lnor | orse | g |
|---|---|---|---|---|---|---|---|---|
| study1 | 16 | 49 | 12 | 53 | 1.442 | 0.366 | 0.430 | 1 |
| study2 | 10 | 30 | 8 | 32 | 1.333 | 0.288 | 0.538 | 1 |
| study3 | 19 | 61 | 14 | 66 | 1.468 | 0.384 | 0.394 | 1 |
| study4 | 80 | 320 | 25 | 375 | 3.750 | 1.322 | 0.241 | 0 |
| study5 | 11 | 29 | 8 | 32 | 1.517 | 0.417 | 0.531 | 0 |
| study6 | 18 | 47 | 16 | 49 | 1.173 | 0.159 | 0.400 | 0 |

or, odds ratio; lnor, log odds ratio; orse, standard error of odds ratio; g, group. Hwang SD & Shim SR[1].

# Supplementary Material 2. R 중재 메타분석 코드

```
#예제자료는 shim_con(Appendix 1), hwang_bin.csv(Appendix 2) 임.

#"meta" 패키지 불러오기
library(meta)
```

## ▶ 연속형(continuous data) 예제자료

```
#데이터 코딩 및 불러오기
data_con <- read.csv("shim_con.csv", header=TRUE)

##종합효과크기 계산##

#원자료로 계산
#SMD를 쓰려면 method.smd="Hedges"(default,보정된 Hedges가 권고됨) or "Cohen",
MD를 쓰려면 smd="MD" 로 표시.
ma_con <- metacont(n1, m1, s1, n2, m2, s2, sm="SMD", method.smd="Hedges",
study,byvar=g ,data=data_con)
print(ma_con, digits=3)

#forest plot
forest(ma_con, comb.fixed=TRUE, comb.random=TRUE,digits=3,rightcols=c("effect", "ci"))
#meta-regression
metareg(ma_con,g,method.tau="REML", digits=3)
bubble(metareg(ma_con,g,method.tau="REML"))

##publication bias##

#funnel plot
funnel(ma_con, comb.fixed=TRUE, comb.random=FALSE)

#출판편향 통계적 검정
#metabias method.bias="linreg"(egger test) / method.bias="rank"(begg test)
ma_con <-
metacont(n1,m1,s1,n2,m2,s2,sm="SMD",method.smd="Hedges",study,data=data_con)
metabias(ma_con, method.bias="linreg")
metabias(ma_con, method.bias="rank")
```

## ▶ 이분형(binary data) 예제자료

```
#데이터 코딩 및 불러오기
data_bin <- read.csv("hwang_bin.csv", header=TRUE)

##종합효과크기 계산##

#원자료로 계산
```

```
ma_bin <- metabin(tp,tp+fp,fn,fn+tn, sm="OR", method ="Inverse", study,byvar=g, data=data_bin)
print(ma_bin, digits=3)

#forest plot
forest(ma_bin, comb.fixed=TRUE, comb.random=TRUE,digits=3,rightcols=c("effect", "ci"))

#meta-regression
metareg(ma_bin,g,method.tau="REML", digits=3)
bubble(metareg(ma_bin,g,method.tau="REML"))

##publication bias##

#funnel plot
funnel(ma_bin, comb.fixed=TRUE, comb.random=FALSE)
#출판편향 통계적 검정
#metabias method.bias="linreg"(egger test) / method.bias="rank"(begg test)
ma_bin <- metabin(tp,tp+fp,fn,fn+tn, sm="OR", method ="Inverse", study, data=data_bin)
metabias(ma_bin, method.bias="linreg", k.min=6)
metabias(ma_bin, method.bias="rank", k.min=6)
```

## ▶ 자료유형 상관없이 메타분석

```
##연속형자료의 효과크기와 표준오차로 계산##
ma_con_es <- metagen(cohen_d, cohen_se, sm="Cohen(SMD)",study,byvar=g, data=data_con)
print(ma_con_es, digits=3)
forest(ma_con_es, comb.fixed=TRUE, comb.random=TRUE,digits=3,rightcols=c("effect", "ci"))

##이분형자료의 효과크기와 표준오차로 계산##
ma_bin_es <- metagen(lnor, orse, sm="OR", study,byvar=g, data=data_bin)
print(ma_bin_es, digits=3)
forest(ma_bin_es, comb.fixed=TRUE, comb.random=TRUE,digits=3,rightcols=c("effect", "ci"))

##유병률자료의 효과크기와 표준오차로 계산##
prop_es <- metagen(p, se, sm="Proportion", backtransf=FALSE,studlab=study,byvar=g, data=data_prop) #untransformed
print(prop_es, digits=3)
forest(prop_es, comb.fixed=TRUE, comb.random=TRUE,digits=3, rightcols=c("effect", "ci"))
prop_es_ln <- metagen(lnp, se, sm="Proportion", backtransf=TRUE, studlab=study, byvar=g, data=data_prop) #ln transformed
print(prop_es_ln, digits=3)
prop_es_logit <- metagen(logitp, se, sm="Proportion", backtransf=TRUE, studlab=study, byvar=g, data=data_prop) #logit transformed
print(prop_es_logit, digits=3)
```
