# Supplementary material for: Intervention meta-analysis: application and practice using R software
Source: Epidemiol Health. 2019 Mar 28;41:e2019008. doi: 10.4178/epih.e2019008 (PMC6545497; doi:10.4178/epih.e2019008)
Supplement: Supplementary file 2 [file epih-41-e2019008-supplementary2.pdf]

Volume: 41, Article ID: e2019008

https://doi.org/10.4178/epih.e2019008

<방법론 단신>

중재 메타분석: R을 활용한 적용과 실제

Intervention Meta-analysis: Application and Practice using R software

심 성 률[1,2], 김 성 장[3,4,5]

[1]고려대학교 의과대학 예방의학교실, [2]순천향대학교 서울병원 비뇨의과학연구소, [3]부산대학교 의과대학 핵의학과, [4]양산부산대학교병원, 의생명융합연구소, [5]양산부산대학교병원, 핵의학과

Sung Ryul Shim, MPH, PhD[1,2], Seong-Jang Kim, MD, PhD[3,4,5]

[1]Department of Preventive Medicine, Korea University College of Medicine, Seoul, Korea, [2]Urological Biomedicine Research Institute, Soonchunhyang University Hospital, Seoul, Korea, [3]Department of Nuclear Medicine, College of Medicine, Pusan National University, Yangsan, Korea, [4]BioMedical Research Institute for Convergence of Biomedical Science and Technology, Pusan National University Yangsan Hospital, Yangsan, Korea, [5]Department of Nuclear Medicine, Pusan National University Yangsan Hospital, Yangsan, Korea

교신저자: 심성률

sungryul.shim@gmail.com

서울시 성북구 안암로 145

Volume: 41, Article ID: e2019008

https://doi.org/10.4178/epih.e2019008

Abstract

The objective of this study was to describe general approaches of intervention meta-analysis that are available for quantitative synthesis of data using R software.

We conducted an intervention meta-analysis using two types of data that included difference in means in continuous data and odds ratio in binary data. The package commands of R software were metacont, metabin, and metagen for overall effect size, forest for forest plot, metareg for meta-regression analysis, and funnel and metabias for publication bias. The estimated overall effect sizes, test for heterogeneity and moderator effect, and the publication bias were reported using R software. Especially authors stressed how to calculate effect sizes of target studies in intervention meta-analysis. This study focused on the practical methods of intervention meta-analysis rather than theoretical concepts for Korean researchers who were non-majored in statistics. Through this study, authors hope that many Korean researchers will use R software to perform an intervention meta-analysis more easily and that related research will be activated.

Keywords: Meta-analysis, Meta-regression, Forest plot, Heterogeneity, Publication bias, R software.

서론

메타분석(meta-analysis)은 체계적·객관적으로 대상문헌을 선택한 후 개별 연구들의 결과를 계량화하여 이를 통합된 효과크기(effect size)로 제시함으로서 근거기반의료(evidence based medicine; EBM)를 위한 올바른 의사결정을 할 수 있게 한다 [1,5].

메타분석을 보다 쉽게 접근하기 위한 컴퓨터 소프트웨어는 STATA, R, SAS, MIX, CMA, RevMan, Meta-Analyst 등이 있다.
RevMan(Review Manager)과 CMA(Comprehensive meta-analysis)는 그래픽 사용자 입력방식으로 초보자가 이용하기에 적합하지만 확장성은 제한적이어서 special topic인 네트워크 메타분석, 진단검사 메타분석, 용량-반응 메타분석, 유전체 메타분석 등은 불가능하다.
반면 R과 STATA는 확장성이 좋아 현재까지 개발되어진 중재 메타분석에서부터 진단검사 메타분석에 이르기까지 다양한 분석이 가능하다.
STATA는 상용 프로그램이며 뛰어난 확장성과 더불어 대부분의 통계 모듈들이 STATA 저널에서 검증을 거치기에 표준적이고 신뢰할 수 있다.
R은 무료 프로그램이며 기본적으로 통계전공자를 위한 프로그래밍 언어를 사용하기 때문에 이를 구현하기에는 상당한 학습이 필요하지만 메타분석 같은 특정 도구로서의 R은 기초적인 패키지 사용과 데이터 및 함수 설정 방법 정도만 익힌다면 누구라도 쉽게 이용할 수 있다. 더욱이 R studio를 같이 설치한다면 그래픽 사용자 입력방식을 지원하기에 매우 편리하다.

본 연구는 저자가 수행했던 메타분석 선행연구들을 [1-3] R 소프트웨어를 이용해서 다시 풀어낸 것이다. 또한 본 연구는 종합효과크기 계산을 위한 효과크기의 유형과

변경부터 다룰 것이기 때문에 메타분석을 실시하기 위한 선행 과정들 – PICO(population, intervention, comparison, outcome)에 기초한 체계적 문헌 수집, 데이터 추출, 그리고 질(quality assessment)평가에 대한 사전 지식이 수반되어야 한다.

효과크기의 이해

메타분석을 수행하려면 가장 먼저 효과크기에 대한 이해가 필요하다. 간단히 말하자면 효과크기란 특정 중재(intervention)에 따른 효과를 나타낸다. 예를 들어 특정 약물 또는 치료가 투입되었다면 이로서 얻게 되는 이익(또는 손해)을 말하며 통상적으로 양적 수치로 표현한다 [1].

연속형 자료(continuous data)에서는 평균의 차이(MD, difference in means), 이분형 자료(binary data) 또는 생존형 자료(time to event data)에서는 위험도비(odds ratio, relative risk, 또는 hazard ratio), 그리고 유병률 자료(proportion or rate data)에서는 백분율(percentage) 형태로 효과크기(effect size)가 표현된다. 그러나 일반적으로 보건의료 메타분석에서는 치료에 따른 직접적인 효과크기를 주요하게 판단하기 때문에 변수간의 상관성을 나타내는 상관계수(correlation coefficient, r)는 효과크기로 잘 쓰여지지 않고 있다.

위험도비의 형태와 백분율은 이미 자료들간의 표준화가 이루어져있기 때문에 이를 효과크기 그대로 사용하여도 큰 문제는 없다.

그러나 연속형 변수의 경우 평균의 차이(MD, difference in means)를 효과크기로 사용하게 되는데 이때는 효과크기의 표준화를 고려해주어야 한다. Cochrane에서는 MD(mean difference) 또는 difference in means로 지칭하며 개별 연구들이 동일한

Volume: 41, Article ID: e2019008

https://doi.org/10.4178/epih.e2019008

단위(scale)일 경우 사용이 가능하며 단위 그대로 이해하면 되기에 해석이 용이하다. WMD(weighted mean difference) 또는 absolute MD가 동일한 용어이다.

표준화된 효과크기 SMD(standardized mean difference)는 개별연구들의 단위가 다를 경우 이들을 표준화해야 상호 비교가 가능하다. 표준화란 효과크기를 표준편차(standard deviation)로 나누는 것이다.

Figure 1은 SMD의 크기를 잘 표현하고 있는데 SMD는 표준정규분포 곡선의 z값에 해당하는 확률 밀도크기와 동일하다. 예를 들어 SMD가 1.96이면 0을 중심으로 양의 방향으로 47.5%에 위치한다. 한가지 주의할 점은 효과크기의 방향에 따라 단방향으로 해석해야 한다. 왜냐하면 참조그룹의 기준점이 0이기 때문이다. 따라서 SMD 가 1.96을 다시 해석하면 '치료그룹은 참조그룹 대비 95% 우수/열등 하다'의 의미가 된다.

1. 효과크기 계산

본 연구에서는 메타분석를 위해서 기본적으로 이해하고 있어야할 효과크기와 표준오차 계산을 다루려고 한다. 프로그램을 사용해서 분석해보면 결국 메타분석은 원자료 자체를 넣어서 계산할 수도 있으며 또한 요약된 효과크기와 표준오차로서도 종합 효과크기를 구할 수 있다는 것을 알 수 있다.

이처럼 개별 연구에서의 효과크기와 표준오차를 계산하는 것은 매우 중요한 부분이며 컴퓨터 프로그램과 더불어 스스로 계산할 수 있어야 추후 이어지는 메타분석 special topic으로의 확장된 접근이 가능하다.

1) 연속형 예제자료

Volume: 41, Article ID: e2019008

https://doi.org/10.4178/epih.e2019008

치료그룹과 대조그룹이 있을 때, m1은 치료그룹의 치료 전 평균(pre_mean1)과 치료 후 평균(post_mean1) 평균의 차이이며 m2는 대조그룹의 전/후 평균 차이이다. 따라서 s1과 s2는 각 m1과 m2의 표준편차이다.

md는 단순히 치료그룹과 대조그룹 평균의 차이이며(m1-m2; 평균차이의 방향은 연구자가 의도하는 방향으로 설정), 공통표준편차(pooled standard deviation, sd)와 공통표준오차(pooled standard deviation, se)는 아래 수식으로 계산한다.

$$\mathrm{sd} = \sqrt{\frac{(\mathrm{n}_1 - 1)\mathrm{s}_1^2 + (\mathrm{n}_2 - 1)\mathrm{s}_2^2}{\mathrm{n}_1 + \mathrm{n}_2 - 2}} \quad , \quad se = sd * \sqrt{\frac{1}{n_1} + \frac{1}{n_2}}$$

표준화된 효과크기 SMD는 md를 공통표준편차로(sd) 나누어준 것이며 (SMD = md / sd), SMD의 표준오차(se)는 공통표준편차(sd)에 각 표본수의 역수를 합산한 제곱근을 곱한 것이다.

이때 만들어지는 SMD는 Cohen의 d이며 표본수가 적을 경우 종합효과크기(overall effect size)를 과대추정하는 경향이 있는데 이를 보정해주기 위해서 Hedges의 g를 사용하기도 한다.

2) 이분형 예제자료

이분형 예제자료는 처치의 유무와 질병 개선의 유무에 따라 흔히 2×2 테이블형태로 표시한다. 이때 처치가 있으면서 질병 개선됨(true positive, tp), 처치가 있으면서 질병이 개선안됨(false positive, fp), 처치가 없으면서 질병이 개선됨(false negative), 처치가 없으면서 질병이 개선안됨(true negative, tn)이라고 하면 효과크기 OR(odds

ratio)과 표준오차(se)는 아래 수식으로 계산한다.

$$\mathrm{OR} = \frac{(\mathrm{tp} * \mathrm{tn})}{(\mathrm{fp} * \mathrm{fn})}\ , \quad se = \sqrt{\frac{1}{\mathrm{tp}} + \frac{1}{\mathrm{fp}} + \frac{1}{\mathrm{fn}} + \frac{1}{\mathrm{tn}}}$$

R의 “meta” 패키지를 이용한 중재 메타분석

Figure 2는 일반적인 중재 메타분석의 흐름을 나타낸다. 최초 자료 코딩시 해당 함수에 적합하도록 변수명을 수정해야하며 메타분석 모델선정(Fixed or Random)으로 종합효과크기를 제시하고 -> 이질성을 확인한 다음 -> 출판 편향을 확인해서 보고한다.

R에서 메타분석을 실시하는 패키지는 크게 “meta”, “metafor”, 그리고 “rmeta”가 있다. 서로간의 장단점이 있으며 필요한 함수를 쓸 수 있으니 미리 설치한다 [6].

```
·install.packages("meta")
·install.packages("metafor")
·install.packaqes("rmeta”)
```

주요 설명은 실행하기 쉬운 “meta” 패키지를 중심으로 기술한다.

본문과의 구분을 위하여 명령어 앞에는 ‘·’으로 표시하였다. 명령어가 길어져서 다음 줄로 넘어가더라도 ‘·’ 없으면 앞의 줄에서 이어지는 것이다. 따라서 실제 R 프로그램에 입력시에는 ‘·’는 제외하고 타이핑하여야 한다.

1. 연속형(continuous data) 예제자료

1) 데이터 코딩 및 불러오기

Volume: 41, Article ID: e2019008

https://doi.org/10.4178/epih.e2019008

척수손상 동물모델에서 줄기세포치료에 따른 방광기능 효과를 메타분석한 연구로서 결과지표는 배뇨 압력(voiding pressure)을 예제로 사용하였다. 전체 연구 수는 11개였고 실험군 94개와 대조군 93개로 이루어졌다. subgroup 1은 contusion model 그리고 0은 transection and hemisection model로 구분하였다 (Supplementary Material 1).

메타분석을 실행하기위해 meta 패키지를 로딩시킨다.

```
·library(meta)
```

다음은 작업폴더에 넣어둔 예제 파일을 아래의 명령어로 R의 메모리에 불러온다. 한가지 주의할 점은 R에서는 쉼표로 구분된 수치파일(csv)의 형태를 선호하니 Appendix 1을 “shim_con.csv” 포멧으로 저장해서 지정된 작업폴더에 넣어 두어야한다.

```
·data_con <- read.csv("shim_con.csv", header=TRUE)
```

read.csv는 csv파일을 불러오는 함수로서 파일명 “shim_con.csv"를 불러와서 파일의 첫번째 변수명을 그대로 쓴다는 뜻이다(header=TRUE). 이렇게 로딩된 파일은 R 메모리에서는 data_con 이름의 데이터로 저장된다.

2) 종합 효과크기

meta 패키지는 하위에 여러 함수들을 포함하는데 그중 metacont 함수는 연속형 자료에서 원자료들이 모두 있을 때 종합효과크기를 계산한다.

```
·ma_con <- metacont(n1, m1, s1, n2, m2, s2, sm="SMD", method.smd=" Hedges", study,
byvar=g ,data=data_con)
·print(ma_con , digits=3)
```

Volume: 41, Article ID: e2019008

https://doi.org/10.4178/epih.e2019008

연속형 자료에서는 치료그룹과 대조그룹(혹은 원하는 방향대로 반대로 입력)의 표본수, 평균, 표준편차를 각각 차례대로 넣어준다.

개별연구들의 단위가 동일하여 효과크기를 표준화하지 않고 계산하려면 smd="MD"로 입력하면 된다. 그러나 통상적으로는 표준화된 효과크기는 SMD로 표시하며 SMD를 산출하는 방법은 여러가지가 있다. 가장 기본적인 방법은 효과크기를 공통표준편차로 나누어주는 Cohen의 d를 말하는 것으로 표본수가 적을 경우 종합 효과크기가 과대추정되는 경향이 있으므로 이를 교정해주는 Hedges의 g를 사용하는 것이 바람직하다(method.smd="Hedges" 또는 "Cohen"). 참고로 Hedges의 g는 교정지수 J를 Cohen의 d에 곱하여 계산한다.

$$J = 1 - \frac{3}{4(n_1 + n_2) - 9}$$

Fixed 또는 Random effect model의 설정을 위해서는 comb.fixed=TRUE 또는 FALSE, comb.random= TRUE 또는 FALSE를 추가해서 입력한다. 만약 모형설정을 하지 않는다면 metacont 함수에서는 두 모형의 결과를 모두 제시한다.

study는 개별 연구들의 이름을 나타내며 data=data_con은 R 메모리에 로딩된 data_con이라는 데이터를 지정해주는 것이다. subgroup별 결과를 나타내려면 byvar=g를 입력하는데 g는 subgroup을 나타내는 변수명이다. metacont 함수를 사용해서 나온 결과들은 ma_con에 지정되며 결과는 Figure 3이다.

ma_con에서 나온 결과들을 Figure 3에서 하나씩 살펴보자.

①은 전체 11개 연구의 종합 효과크기를 나타낸다. Fixed effect model의 SMD는 -1.456 (95%CI; -1.832, -1.081) *p*-value <0.0001이하로서 해당 처치가 통계적으로 유의

https://doi.org/10.4178/epih.e2019008

하게 개선되는 결과를 나타내었다. Random effect model의 SMD는 -1.973 (95%CI; -2.897, -1.048) *p*-value <0.0001이하로서 동일한 결과를 나타낸다.

②와 ③은 subgroup에 해당하는 결과를 Fixed or Random effect model로서 나타낸 것이다. Random 모델에서는 subgroup(0 vs 1)에 따른 차이가 의심된다.

④는 전체 연구의 이질성(heterogeneity)를 나타낸 것이다. 이질성의 Higgins'I^2는 Cochrane Q statistics에서 자유도(degree of freedom)를 뺀 것을 다시 Cochrane Q statistics으로 나누어 준 값으로 이질성을 일관성 있게 정량화시킨다. 0%에서 40%는 이질성이 중요하지 않을 수 있으며 (might not be important), 30%에서 60%는 중간 이질성(moderate heterogeneity), 50%에서 90%는 중대한 이질성(substantial heterogeneity), 그리고 75%에서 100%는 심각한 이질성(considerable heterogeneity)을 나타낸다. Cochrane Q statistics의 *p*-value는 조금 폭 넓게 0.1을 유의성 판단 기준으로 한다 [4].

본 연속형 예제자료의 Higgins'I^2는 82.7%이며 Cochrane Q statistics *p*-value <0.0001 이하로서 이질성이 존재한다는 것을 알 수 있다. 따라서 전체적인 모델은 Random effect model을 우선하여야 한다.

그 외 Figure 3 하단에 해당 결과가 어떤 계산방법으로 도출되었는지 보여주고 있다. Inverse variance method는 메타분석의 기본적인 방법으로서 개별 연구들의 가중치를 계산할 때 해당 연구의 역분산을 활용한다. DerSimonian-Lair estimator는 Random effect model에서 연구간 변량을 계산할 때 tau값을 계산하는 방법이며, Hedges'g는 현재의 결과값은 Cohen's d를 보정한 Hedges의 g를 사용하였다는 것이다.

세부적인 계산방법은 meta 패키지를 참조하면서 임의대로 자유롭게 설정할 수 있

Volume: 41, Article ID: e2019008

https://doi.org/10.4178/epih.e2019008

다.

■ Forest plot(숲 그림)

Figure 3은 상세한 정보파악은 좋으나 전반적인 식별력이 떨어진다. 따라서 forest plot을 작성해줌으로서 독자들의 이해도를 향상시킬 수 있다 (Figure 4).

·forest(ma_con, comb.fixed=TRUE, comb.random=TRUE,digits=3,rightcols=c("effect", "ci"))

forest 함수에 해당 설정된 메타분석 모델(ma_con)을 입력한다. 그런 다음 다양한 옵션들을 넣어주어 그림을 예쁘게 만들어준다. comb.fixed=TRUE와 comb.random=TRUE는 두 모형을 다 표시하라는 것이며, digits=3은 소수점 세자리까지만 표시, rightcols=c(“effect”,”ci”))는 forest plot 오른편에는 원래는 weight가 표시되는데 이를 생략하고 효과크기와 신뢰구간만을 보여주라는 뜻이다.

Figure 4는 앞의 종합 효과크기와 동일한 정보를 제공한다. 더불어 개별연구들의 효과크기를 그래픽으로 제시함으로써 연구 내 변동과 연구 간 변동을 쉽게 파악할 수 있도록 해준다.

예를 들어 연구 내 변량이 큰 것은 Mitsui2005_a 그리고 Mitsui2003인 것을 알 수 있고 연구 간 변량이 큰 것은 Mitsui2005_a, WBPark2010_1 그리고 WBPark2010_2인 것을 알 수 있다.

3) 이질성 (heterogeneity)

메타분석에서 얻어낸 종합 효과크기를 제대로 해석하려면 연구들간의 이질성 유무를 확인하고 만약 유의한 조절변수(moderator)가 있다면 이를 검정하고 보고하여야

한다. 이러한 이질성의 원인은 우연(chance), 연구설계(study design)의 차이, 연구환경, 그리고 표본집단의 인구사회학적 요인에 이르기까지 매우 다양하다.

(1) 시각적확인: forest plot & subgroup analysis

이질성을 탐색하기 위해 연구내 변동과 연구간 변동을 시각적으로 쉽게 확인 할 수 있다.

(2) 이질성 측정: Higgins'I^2 & Cochrane Q statistics

Figure 3을 설명한면서 연구의 이질성을 상세 설명하였고 이질성의 정도를 수치화해서 나타내며 더불어 통계적 검정도 보여준다.

(3) 이질성 원인파악 : meta-regression

Forest plot을 이용한 시각적 확인과 Cochrane Q statistics와 Higgins' I^2 이용한 이질성 수치로부터 이질성이 의심된다면 이질성의 원인을 통계적으로 검정하기 위한 파악하기 위하여 메타회귀분석을 실시한다.

·metareg(ma_con,g, method.tau="REML", digits=3)

metareg 함수에 설정된 메타분석 모델을 넣어주고, 메타회귀분석에 가중치를 부여하는 방법에 따라 method.tau="REML"(restricted maximum-likelihood estimator or "ML"(maximum-likelihood estimator), "DL"(DerSimonian-Laird estimator) 등을 선택한다. 가중치 계산방법에 따른 수치의 변화는 있지만 대부분의 통계적 방향성은 동일하니 너무 주의를 기울이지 않아도 무방하다.

Random effect model을 기준으로 subgroup 1의 종합효과크기는 -2.139(95%CI; -3.410, -0.867) 그리고 subgroup 0의 종합효과크기는 -1.610(-2.413, -0.808)로 해당 변수가 조절변수(moderator)로 의심되었으나 메타회귀분석 결과 p-value = 0.711 로

Volume: 41, Article ID: e2019008

https://doi.org/10.4178/epih.e2019008

통계적으로 유의한 차이를 나타내지는 않았다.

·bubble(metareg(ma_con, g, method.tau="REML"))

메타회귀분석결과를 Figure 5에서와 같이 도식화해서 나타낼 수 있다. 그래프상의 직선은 회귀직선을 나타내며 그 기울기에 대한 통계적 검정이 앞서 실시한 메타회귀분석의 *p*-value이다.

4) 출판편향 확인 (publication bias)

출판편향(publication bias)은 개별 연구들의 특성과 결과에 따라 연구가 출판되거나 출판되지 않을 오류이다. 일반적으로 통계적 유의한 연구결과일 경우 더욱 출판될 가능성이 높기 때문에 발생하는데, 이러한 출판편향을 고려하여 해당 메타연구의 결과가 과대 또는 과소 추정되지는 않았는지 확인하여야 한다.

(1) 시각적확인: funnel plot

출판편향을 탐색하기 위해 연구들간의 비대칭성이 존재하는지 시각적으로 확인하여야 한다 (Figure 6).

·funnel(ma_con, comb.fixed=TRUE, comb.random=FALSE)

funnel 함수에 설정된 메타분석 모델을 넣고, comb.fixed=TRUE 또는 FALSE, comb.random= TRUE 또는 FALSE를 추가해서 입력한다.

Funnel plot의 Y축은 표본크기(표준오차)를 X축은 효과크기를 제시한다. 일반적으로 작은 규모의 연구들은 아래쪽에 넓게 분포되며 큰 규모의 연구들은 깔때기 안 상단에 좁게 분포된다. 따라서 깔때기 안 상단에 좌우대칭으로 골고루 분포되어 있다면

출판편향은 적다고 판단할 수 있다.

연속형 예제자료는 깔때기 바깥 좌측으로 3개, 우측으로 2개의 연구가, 깔때기 안 좌측으로 4개, 우측으로 2개의 연구가 분포한다. 시각적으로 판단하기에 출판편향이 있을 것으로 판단된다 (Figure 6).

(2) 출판편향 통계적 검정

출판편향을 통계적으로 검정하는 일반적인 방법은 Egger’s linear regression method test (Egger’s test) 그리고 Begg and Mazumdar’s rank correlation test (Begg’s test)가 있다. Egger’s test가 Begg’s test보다 효과크기의 실제 추정치를 더 정확히 추정한다고 보고하고 있다. 그러나 통계적 검정은 출판편향의 영향보다는 연구의 수가 적을 경우(small study effect) 이를 정확히 검정하지 못하므로 Cochrane에서는 권고하지 않는다.

■ Egger’s linear regression method test

중재효과의 표준오차에 대한 개별 연구들의 효과크기 관계를 회귀식으로 나타낸 것으로 귀무가설은 회귀식의 초기값(intercept)은 우연에 의한 결과로서 출판편향이 있음을 증명할 수 없다는 것이다.

```
·ma_con <- metacont(n1, m1, s1, n2, m2, s2, sm="SMD", method.smd="Hedges", study,data=data_con)
·metabias(ma_con, method.bias="linreg")
```

앞선 종합효과크기 계산에서 subgroup 분석으로 나누었기에 metabias 함수가실행되지 않을 가능성이 크다. 따라서 전체 연구를 대상으로 다시한번 종합효과크기를 계산한 다음 바로 이어서 metabias 함수를 사용한다. metabias 함수에 설정된 메타분석 모델과 Egger’s test를 실행하는 method.bias="linreg" 옵션을 추가한다.

Volume: 41, Article ID: e2019008

https://doi.org/10.4178/epih.e2019008

연속형 자료의 경우 bias항목의 Coef.가 -9.23으로 초기값(intercept)을 나타내며 해당 *p*-value <0.0001로써 귀무가설을 기각하여 출판편향이 있음을 확인할 수 있다.

■ Begg and Mazumdar's rank correlation test

개별 연구들의 표준화된 효과크기와 표준오차와의 상관관계를 보정된 순위상관(rank correlation)으로 검정한다. 순위상관 검정이 유의하지 않다면 출판편향이 없음을 나타낸다.

·metabias(ma_con, method.bias="rank")

metabias 함수에 설정된 메타분석 모델과 Begg's test를 실행하는 method.bias="rank" 옵션을 추가한다.

Egger's test 결과와 마찬가지로 *p*-value = 0.0024로서 출판편향이 있음을 알 수 있다.

이처럼 출판편향이 통계적으로 유의할 때는 출판편향이 의심되는 연구들을 포함 또는 제외하여 종합효과크기를 다시한번 확인할 필요가 있다. 즉, 출판 편향에 대한 민감도 분석(sensitivity analysis)을 실시하여 해당 연구들의 특성을 보고하고 이질성이 발견된다면 메타회귀분석을 통해서 통계적 검정도 실시하여야 한다.

2. 이분형(binary data) 예제자료

메타분석을 실행을 위한 명령어는 연속형 예제자료와 대부분 동일하므로 차이가 있는 부분을 중심으로 설명하겠다.

1) 데이터 코딩 및 불러오기

전체 연구 수는 6개였고 전체 표본수는 1,380으로 이루어졌다. g는 subgroup 분석을 위해 임의로 0과 1을 설정하였다 (Supplementary Material 1).

Supplementary Material 1의 “hwang_bin.csv” 포멧으로 저장해서 지정된 작업폴더에 넣어 두어야한다.

```
·data_bin <- read.csv("hwang_bin.csv", header=TRUE)
```

2) 종합 효과크기

meta 패키지는 하위에 여러 함수들을 포함하는데 그중 metabin 함수는 이분형 자료에서 원자료들이 모두 있을 때 종합효과크기를 계산한다.

```
·ma_bin <- metabin(tp,tp+fp,fn,fn+tn, sm="OR", method ="Inverse", study, byvar=g, data=data_bin)
·print(ma_bin, digits=3)
```

이분형 자료에서는 tp, tp+fp, fn, fn+tn을 각각 차례대로 넣어준다.

효과크기를 OR 또는 RR로 표시하고 싶으면 sm=“OR” 또는 “RR”로 설정한다. 개별 연구들의 가중치를 설정하는 방법이 다수 있는데 일반적인 inverse variance method를 사용하려면 method=“Inverse”를 입력한다.

metabin 함수를 사용해서 나온 결과들은 ma_bin에 지정되며 Figure 7에서 하나씩 살펴보자.

①은 전체 6개 연구의 종합 효과크기를 나타낸다. Fixed effect model의 OR는 2.063 (95%CI; 1.526, 2.789) *p*-value <0.0001이하로서 동아리 활동이 취업에 통계적으로 유의하게 영향을 미치는 결과를 나타내었다. Random effect model의 OR은 1.762 (95%CI; 1.103, 2.813) *p*-value = 0.0177로서 동일한 결과를 나타낸다.

Volume: 41, Article ID: e2019008

https://doi.org/10.4178/epih.e2019008

②와 ③은 subgroup에 해당하는 결과를 Fixed or Random effect model로서 나타낸 것이다. Random 모델에서는 subgroup(0 vs 1)에 따른 차이가 의심된다.

④는 전체 연구의 이질성(heterogeneity)를 나타낸 것이다.

본 이분형 예제자료의 Higgins'I^2는 52.6%이며 Cochrane Q statistics *p*-value = 0.061로서 이질성이 있음을 알 수 있다.

그외 Figure 7 하단에 해당 결과가 어떤 계산방법으로 도출되었는지 밝히고 있다. Inverse variance method는 메타분석의 기본적인 방법으로서 개별 연구들의 가중치를 계산할 때 해당 연구의 역분산을 활용한다. DerSimonian-Lair estimator는 Random effect model에서 연구간 변량을 계산할 때 tau값을 계산하는 방법이다.

세부적인 계산방법은 meta 패키지를 참조하면서 임의대로 자유롭게 설정할 수 있다.

■ Forest plot(숲 그림)

```
·forest(ma_bin, comb.fixed=TRUE, comb.random=TRUE,digits=3,rightcols=c("effect", "ci"))
```

forest 함수에 해당 설정된 메타분석 모델(ma_bin)을 입력한다. 상세 옵션 설명은 연속형 예제자료와 동일하다.

3) 이질성 (heterogeneity)

(1) 이질성 원인파악 : meta-regression

Forest plot을 이용한 시각적 확인과 Cochrane Q statistics와 Higgins' I^2 이용한 이질성 수치로부터 이질성이 의심된다면 이질성의 원인을 통계적으로 검정하기 위한 파악하기 위하여 메타회귀분석을 실시한다.

Volume: 41, Article ID: e2019008

https://doi.org/10.4178/epih.e2019008

·metareg(ma_bin, g, method.tau="REML", digits=3)

Random effect model을 기준으로 subgroup 1의 종합효과크기는 1.428(95%CI; 0.865, 2.357) 그리고 subgroup 0의 종합효과크기는 2.542(1.743, 3.707)로 해당 변수가 조절변수(moderator)로 의심되었으나 메타회귀분석 결과 *p*-value = 0.371 로 통계적으로 유의한 차이를 나타내지는 않았다.

메타회귀분석을 위해서는 대상 연구가 최소 10개 이상은 있어야 유의미한 결과로 해석할 수 있다. 그러나 본 이분형 예제자료는 전체 연구의 수가 6개로 메타회귀분석에 적절하지 않고 통계적 유의차를 나타내지 못하였으나 추후 연구의 수가 추가된다면 해당 조절변수는 유의하게 영향을 미칠 것으로 판단된다.

4) 출판편향 확인 (publication bias)

(1) 시각적확인: funnel plot

·funnel(ma_bin, comb.fixed=TRUE, comb.random=FALSE)

이분형 예제자료의 funnel plot을 그려보면 깔때기 안 좌측으로 4개, 깔때기 바깥 우측으로 1개의 연구가 분포한다. 시각적으로 판단하기에 출판편향이 있을 것으로 판단된다. 따라서 연속형 예제에서 실시하였던 출판편향의 통계적 검정을 이용하여 고찰하기 바란다.

3. 자료유형 상관없이 메타분석

Volume: 41, Article ID: e2019008

https://doi.org/10.4178/epih.e2019008

지금까지 연속형(continuous data)과 이분형(binary data)의 원자료에서 종합효과크기와 이와 관련된 이질성을 평가하는 방법을 알아보았다.

그러나 사실 이러한 자료에 따른 구분은 사용자의 편의를 위해서 명령어(함수)를 구분해 놓았을 뿐 개별 연구들의 효과크기와 표준오차를 이미 알고 있다면 자료의 유형에 상관없이 메타분석을 실시할 수 있다.

1) 데이터 코딩 및 불러오기

앞에서 실행했던 연속형(continuous data, Appendix 1)과 이분형(binary data, Appendix 2)의 원자료를 불러오면 효과크기와 표준오차는 이미 변수로 입력되어져 있다.

```
·data_con <- read.csv("shim_con.csv", header=TRUE)
·data_bin <- read.csv("hwang_bin.csv", header=TRUE)
```

read.csv 함수로 연속형과 이분형 자료를 각각 불러들여 R 메모리에서 data_con, data_bin 이름의 데이터로 저장한다.

2) 종합 효과크기

meta 패키지는 하위에 여러 함수들을 포함하는데 그중 metagen 함수는 효과크기와 표준오차로서 종합효과크기를 계산한다.

(1) 연속형 자료 효과크기와 표준오차 계산

```
·ma_con_es <- metagen(cohen_d, cohen_se, sm="Cohen(SMD)", study, byvar=g, data=data_con)
·print(ma_con_es, digits=3)
·forest(ma_con_es, comb.fixed=TRUE, comb.random=TRUE, digits=3, rightcols=c("effect", "ci"))
```

metagen 함수에 효과크기에 해당하는 cohen_d와 표준오차 cochen_se를 입력한다.

Volume: 41, Article ID: e2019008

https://doi.org/10.4178/epih.e2019008

연속형 자료의 효과크기와 표준오차로 산출한 메타분석 모델이 ma_con_es에 설정된다.

앞서 실시한 연속형 예제자료에서 SMD 중 Cohen의 d를 옵션으로 [method.smd="Cohen”] 사용한다면 지금 산출한 메타분석 모델 ma_con_es과 동일한 효과크기를 얻을 수 있다.

(2) 이분형 자료 효과크기와 표준오차 계산

```
·ma_bin_es <- metagen(lnor, orse, sm="OR", study, data=data_bin)
·print(ma_bin_es, digits=3)
·forest(ma_bin_es, comb.fixed=TRUE, comb.random=TRUE, digits=3, rightcols=c("effect",
"ci"))
```

metagen 함수에 효과크기에 해당하는 lnor과 표준오차 orse를 입력한다. 이분형 자료의 효과크기와 표준오차로 산출한 메타분석 모델이 ma_bin_es에 설정된다.

앞서 실시한 이분형 예제자료의 효과크기와 동일함을 알 수 있을 것이다.

맺음말

본 연구는 통계학을 전공하지 않은 일반 연구자들도 쉽게 수행할 수 있도록 통계학 이론을 최소화하여 메타분석의 실질적 수행방법에 집중하였다.

따라서 본 연구를 참고하여 메타분석을 실행하고자 하는 연구자는 반드시 효과크기 계산에 대한 개념 정립을 확고히 하여야 한다. 아울러 국내 연구자들이 R을 이용한 메타분석을 보다 쉽게 수행함으로써 관련 연구가 활성화되기를 바란다.

Volume: 41, Article ID: e2019008

https://doi.org/10.4178/epih.e2019008

References

1. Hwang SD & Shim SR. Meta-analysisp; from forest plot to network meta-analysis. Seoul: Hannarae publishing co.; 2018 (Korean). ISBN: 9788955662214

2. Shim SR. Intervention meta-analysis using R software. Gyeonggi-do: SDB Lab; 2019 (Korean). ISBN 979-11-965933-0-8

3. Shim SR, Shin IS, Bae JM. Intervention Meta-Analysis Using STATA Software. J Health Info Stat. 2016;41(1):123-134.

4. Higgins JPT, Green S. editors Cochrane handbook for systematic reviews of interventions. Version 5.1.0:6.4.1. 2011. The Cochrane Collaboration. Available from: http://www.cochrane-handbook.org.

5. Borensein M, Hedges LV, Higgins JPT, Rothstein HR. (2019). Introduction to Meta-Analysis.preface. West Sussex, UK: John Wiley & Sons Ltd.; p. 20-28.

6. R software "meta" packages. Available from: http://www.imbi.uni-freiburg.de/lehre/lehrbuecher/meta-analysis-with-r/r-packages or http://meta-analysis-with-r.org/

Volume: 41, Article ID: e2019008

https://doi.org/10.4178/epih.e2019008

Figure legends

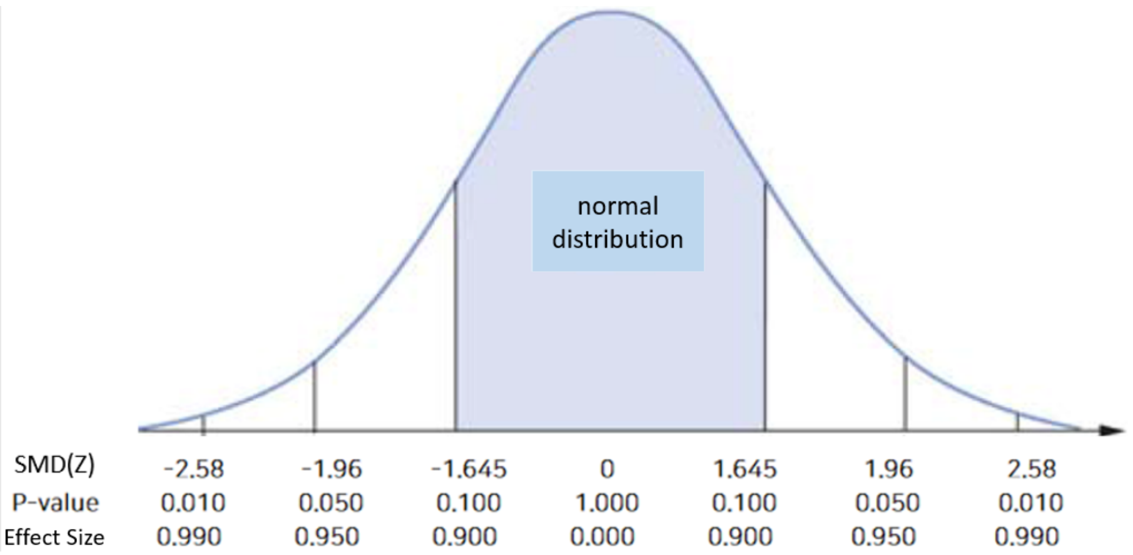

Figure 1. Effect size of standardized mean difference.

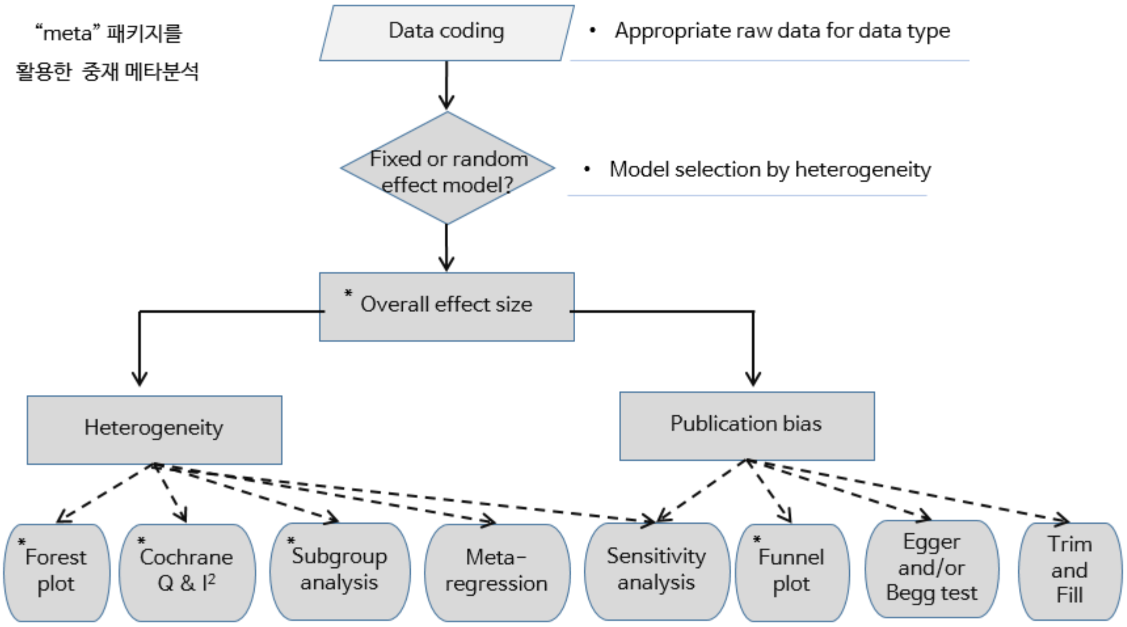

Figure 2. Flow chart of intervention meta-analysis using R "meta" package.

Volume: 41, Article ID: e2019008

https://doi.org/10.4178/epih.e2019008

```
                     SMD            95%-CI %W(fixed) %W(random) g
Mitsui2003       -2.5588 [-4.2551; -0.8625]      4.9        8.2 1
Mitsui2005a      -6.3696 [-8.5509; -4.1884]      3.0        6.9 1
Mitsui2005b      -3.2738 [-4.7997; -1.7479]      6.1        8.7 1
Mitsui2011       -3.3254 [-4.8158; -1.8350]      6.3        8.8 1
Telmeltas2009_1  -2.4536 [-3.9361; -0.9710]      6.4        8.8 0
Telmeltas2009_2  -0.9672 [-2.1393;  0.2049]     10.3        9.6 0
WBPark2010_1      0.0230 [-0.8580;  0.9040]     18.2       10.3 1
WBPark2010_2      0.3247 [-0.7171;  1.3666]     13.0        9.9 1
YHu2012          -1.6858 [-2.8745; -0.4971]     10.0        9.5 0
YingJin2011_1    -1.8309 [-2.9438; -0.7180]     11.4        9.7 1
YingJin2011_2    -1.3869 [-2.5405; -0.2333]     10.6        9.6 1

Number of studies combined: k = 11

 ①                          SMD            95%-CI     z  p-value
Fixed effect model   -1.4565 [-1.8318; -1.0811] -7.60 < 0.0001
Random effects model -1.9729 [-2.8973; -1.0485] -4.18 < 0.0001

Quantifying heterogeneity: ④
tau^2 = 1.9634; H = 2.40 [1.83; 3.14]; I^2 = 82.7% [70.3%; 89.9%]

Test of heterogeneity:
     Q d.f.  p-value
 57.66   10 < 0.0001

Results for subgroups (fixed effect model): ②
        k     SMD            95%-CI     Q  tau^2   I^2
g = 1   8 -1.4066 [-1.8448; -0.9683] 55.06 2.8537 87.3%
g = 0   3 -1.5939 [-2.3211; -0.8666]  2.41 0.0870 17.1%

Test for subgroup differences (fixed effect model):
                   Q d.f.  p-value
Between groups  0.19    1   0.6655
Within groups  57.48    9 < 0.0001

Results for subgroups (random effects model): ③
        k     SMD            95%-CI     Q  tau^2   I^2
g = 1   8 -2.1385 [-3.4101; -0.8669] 55.06 2.8537 87.3%
g = 0   3 -1.6101 [-2.4126; -0.8076]  2.41 0.0870 17.1%

Test for subgroup differences (random effects model):
                    Q d.f. p-value
Between groups   0.47    1  0.4909

Details on meta-analytical method:
- Inverse variance method
- DerSimonian-Laird estimator for tau^2
- Hedges' g (bias corrected standardised mean difference)
```

Figure 3. Overall effect size of continuous example.

Volume: 41, Article ID: e2019008

https://doi.org/10.4178/epih.e2019008

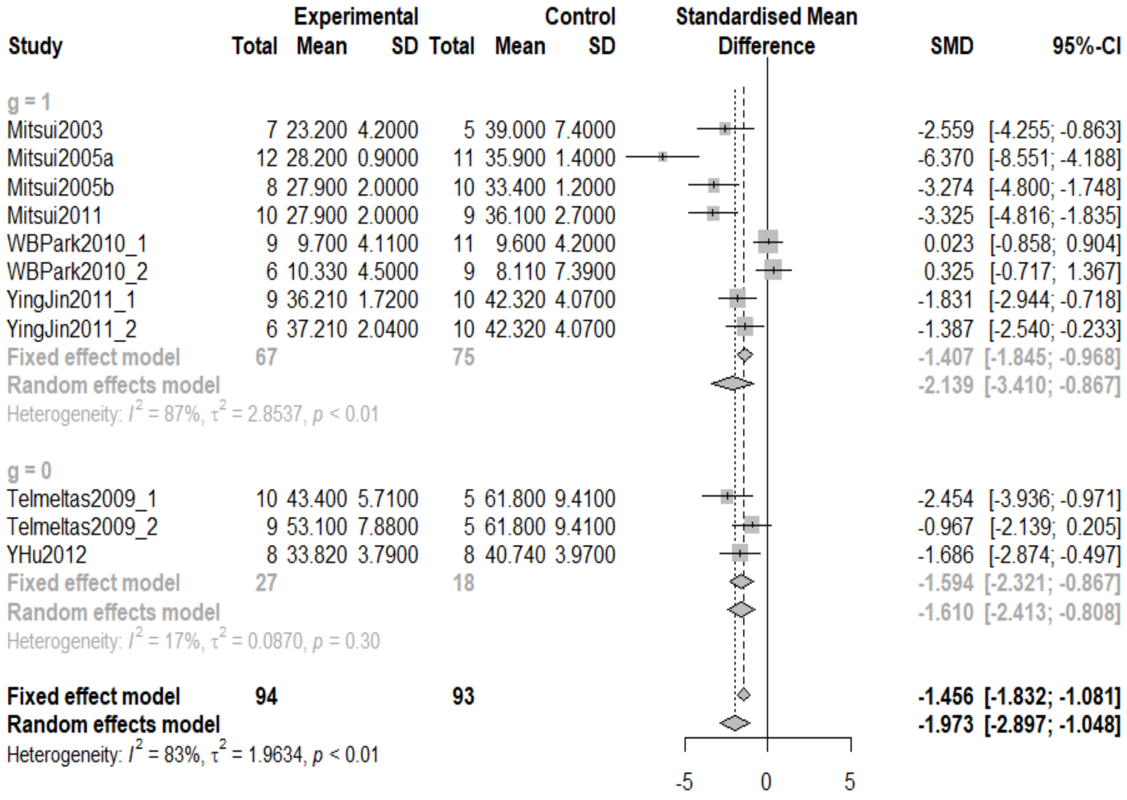

Figure 4. Forest plot of continuous example.

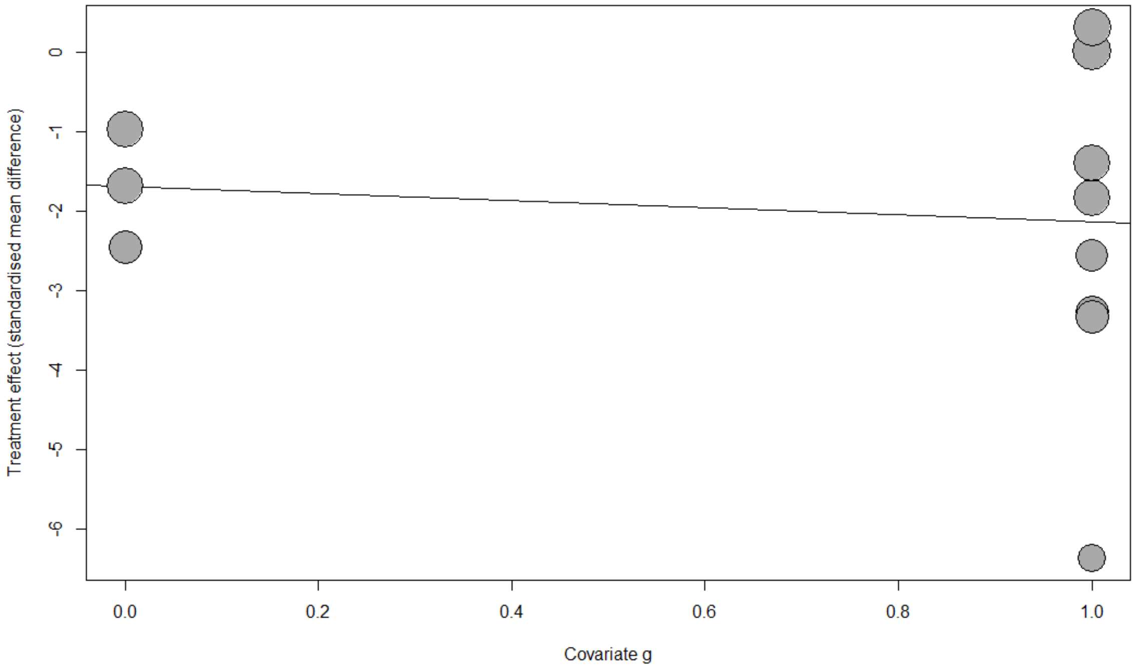

Figure 5. Meta-regression bubble plot of continuous example. .

Volume: 41, Article ID: e2019008

https://doi.org/10.4178/epih.e2019008

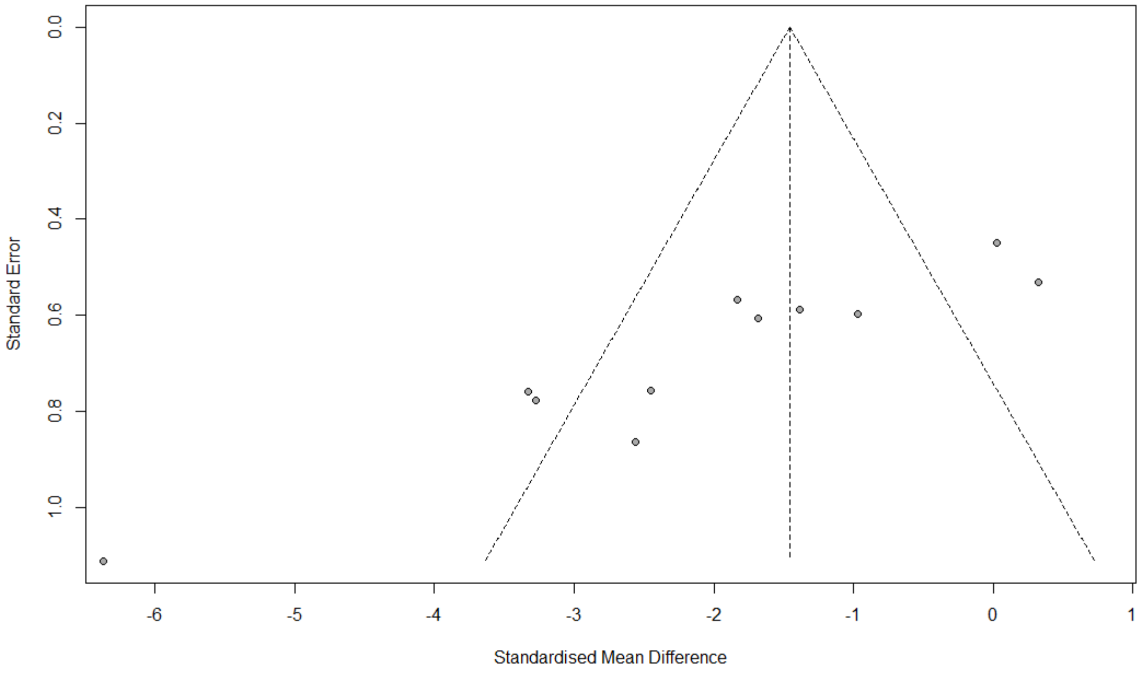

Figure 6. Funnel plot of continuous example.

Volume: 41, Article ID: e2019008

https://doi.org/10.4178/epih.e2019008

```
             OR           95%-CI %W(fixed) %W(random) g
study1 1.442 [0.621; 3.352]      12.8       15.9 1
study2 1.333 [0.464; 3.828]       8.2       12.3 1
study3 1.468 [0.678; 3.181]      15.2       17.4 1
study4 3.750 [2.336; 6.019]      40.6       24.7 0
study5 1.517 [0.536; 4.293]       8.4       12.5 0
study6 1.173 [0.536; 2.567]      14.8       17.1 0

Number of studies combined: k = 6
 ①
                         OR           95%-CI    z  p-value
Fixed effect model    2.063 [1.526; 2.789] 4.71 < 0.0001
Random effects model 1.762 [1.103; 2.813] 2.37    0.0177

Quantifying heterogeneity: ④
tau^2 = 0.1729; H = 1.45 [1.00; 2.30]; I^2 = 52.6% [0.0%; 81.1%]

Test of heterogeneity:
     Q d.f.  p-value
 10.55    5   0.0610

Results for subgroups (fixed effect model): ②
        k    OR           95%-CI    Q  tau^2    I^2
g = 1    3 1.428 [0.865; 2.357] 0.02      0   0.0%
g = 0    3 2.542 [1.743; 3.707] 7.28 0.3739 72.5%

Test for subgroup differences (fixed effect model):
                  Q d.f.  p-value
Between groups 3.25     1   0.0716
Within groups  7.31     4   0.1206

Results for subgroups (random effects model): ③
        k    OR           95%-CI    Q  tau^2    I^2
g = 1    3 1.428 [0.865; 2.357] 0.02      0   0.0%
g = 0    3 2.012 [0.886; 4.567] 7.28 0.3739 72.5%

Test for subgroup differences (random effects model):
                    Q d.f.  p-value
Between groups    0.49     1   0.4844

Details on meta-analytical method:
- Inverse variance method
- DerSimonian-Laird estimator for tau^2
```

Figure 7. Overall effect size of binary example.
